# Morphological and structural characterization of single-crystal ZnO nanorod arrays on flexible and non-flexible substrates

**DOI:** 10.3762/bjnano.6.73

**Published:** 2015-03-12

**Authors:** Omar F Farhat, Mohd M Halim, Mat J Abdullah, Mohammed K M Ali, Nageh K Allam

**Affiliations:** 1 Nano-Optoelectronics Research and Technology Laboratory, School of Physics, Universiti Sains Malaysia, 11800 Penang, Malaysia; 2 Energy Materials Laboratory (EML), Physics Department, School of Sciences and Engineering, The American University in Cairo, New Cairo 11835, Egypt

**Keywords:** chemical bath deposition (CBD), nanorods, Raman, XRD, ZnO

## Abstract

We report a facile synthesis of zinc oxide (ZnO) nanorod arrays using an optimized, chemical bath deposition method on glass, PET and Si substrates. The morphological and structural properties of the ZnO nanorod arrays were investigated using various techniques such as field emission scanning electron microscopy (FESEM) and X-ray diffraction (XRD) measurements, which revealed the formation of dense ZnO nanorods with a single crystal, hexagonal wurtzite structure. The aspect ratio of the single-crystal ZnO nanorods and the growth rate along the (002) direction was found to be sensitive to the substrate type. The lattice constants and the crystallite size of the fabricated ZnO nanorods were calculated based on the XRD data. The obtained results revealed that the increase in the crystallite size is strongly associated with the growth conditions with a minor dependence on the type of substrate. The Raman spectroscopy measurements confirmed the existence of a compressive stress in the fabricated ZnO nanorods. The obtained results illustrated that the growth of high quality, single-crystal ZnO nanorods can be realized by adjusting the synthesis conditions.

## Introduction

Metal oxides are multifunctional materials with a wide range of applications encompassing photonic devices, high-K dielectrics, sensors, implants, and solar cells [1–2]. It is currently perceived that nanoscale control of metal oxide architectures can be used to enhance their performance in these applications. In particular, zinc oxide (ZnO) can be considered the most important among all valve metal oxides. ZnO has been fabricated as nanowires, nanorods, nanoparticles, and nanoneedles, among many other forms [3–5]. However, the majority of the resulting structures are amorphous and require high-temperature heat treatment to induce crystallinity. The need for heat treatment limits their use with temperature-sensitive materials, such polymeric photocatalytic membranes. Consequently, low-temperature fabrication routes are essential to maximize the benefits of the unique material architecture. The crystallinity of the ZnO nanorods must also be controlled for their application in photocatalysis and in dye-sensitized solar cells. Further, single crystals are superior to polycrystalline architectures in a unique way: the decrease of the number of grain boundaries ameliorates charge-carrier transport by permitting a direct and quick charge transport pathway and thus decreases the carrier path length, which in turn decreases recombination losses.

To this end, various methods have been reported in the literature to produce amorphous and polycrystalline ZnO nanomaterials, especially in the form of nanorods. Also, several deposition methods have been reported to fabricate single-crystal ZnO nanorods, such as RF and DC sputtering [6], chemical vapor deposition (CVD) [7], molecular beam epitaxy (MBE) [8], pulsed laser deposition (PLD) [9], vapor phase transport (VPT) [10], and thermal evaporation [11]. However, these methods are considered to be high-cost techniques since they require complex, expensive equipment, high vacuum conditions and high operation temperatures. Further, the fabrication conditions are not compatible with some substrates such as organic materials for flexible and wearable electronics. In contrast, the chemical bath deposition (CBD) method is a low-cost method, requiring low-temperature operation conditions that are suitable for large scale fabrication of ZnO nanoarchitectures on any substrate [12–14].

Herein, we report an optimized CBD method, employed to fabricate single-crystal ZnO nanorod arrays on flexible and non-flexible substrates at low temperature. High quality, oriented ZnO nanorods of uniform thickness and length distribution ensure a desired light absorption and propagation characteristics as well as percolation pathways for charge transfer.

## Experimental

ZnO nanorod arrays were grown on three different substrates (glass, PET and Si) by the chemical bath deposition (CBD) technique. The substrates (2 × 2 cm) were cleaned following the procedures reported elsewhere [15]. All chemicals were of analytical grade and were used as obtained from Aldrich without further purification. Briefly, the substrates were coated with 130 nm of ZnO seed layers using RF sputtering and annealed at 200 °C for 1 h. In a typical procedure, 0.05 M zinc nitrate (Zn(NO_3_)_2_·6H_2_O) was mixed with hexamethylenetetramine (HMT) in a glass beaker and slowly stirred until complete dissolution was achieved. The growth temperature and time was 95 °C and 3 h, respectively. The beaker was then left inside the oven for 30 min to cool down to 40 °C. Finally, the substrates with the grown ZnO nanorods were rinsed in deionized water and dried under N_2_ flow.

The prepared ZnO nanorods were investigated by using different techniques. The thicknesses of the films were determined for each sample from the cross-sectional FESEM images. The crystal structure of the films was evaluated by high-resolution XRD system (PAnalyticalX’pert, PRO MRD PW3040, Netherlands) with a Cu Kα radiation source of λ = 1.5406 Å. For the high-resolution measurements, this system resolution was 12 arc/s. An integrated micro-photoluminescence and a Raman spectrometer (Jobin Yvon, HR800UV) with an argon ion laser source (514.5 nm) were used. The incident laser power was 20 mW. The grating and the hole size were usually set at 50 µm. The Raman scattering experiments were carried out at room temperature with a system resolution of 1 cm^−1^. The surface morphology of the films was studied using FESEM (FESEM; Nova Nano SEM 450, FEI, Japan) with an integrated energy-dispersive X-ray (EDX) unit for the analysis of the chemical composition of the samples.

## Results and Discussion

**Figure 1:**
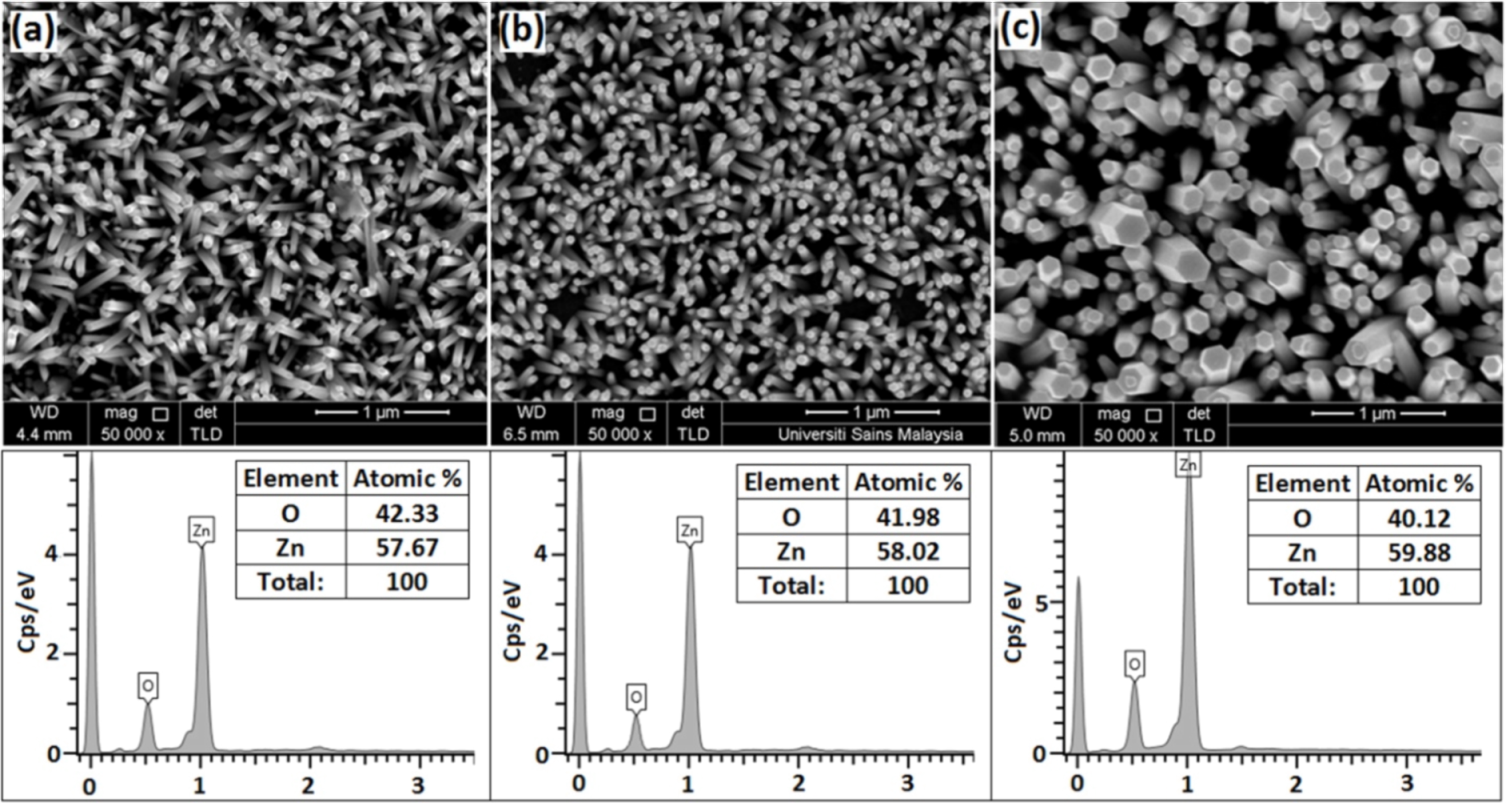
FESEM images and EDX spectrum of the ZnO nanorods grown on (a) glass, (b) PET and (c) Si substrates at 95 °C for 3 h, respectively.

Figure 1a–c shows the FESEM images of the fabricated ZnO nanorod arrays grown on glass, PET and Si substrates, respectively. The nanorods were uniformly grown on all substrates with a hexagonal prism shape, suggesting growth along the (002) direction. Note that the density of nanorods per area decreases from PET to glass to Si substrates. The corresponding EDX analysis reveals the existence of Zn and O, which corresponds to the characteristic composition of ZnO, without the presence any impurities or substrate signal. The ratio between Zn and O was the same for all analyzed samples grown on PET, glass and Si substrates.

**Figure 2:**
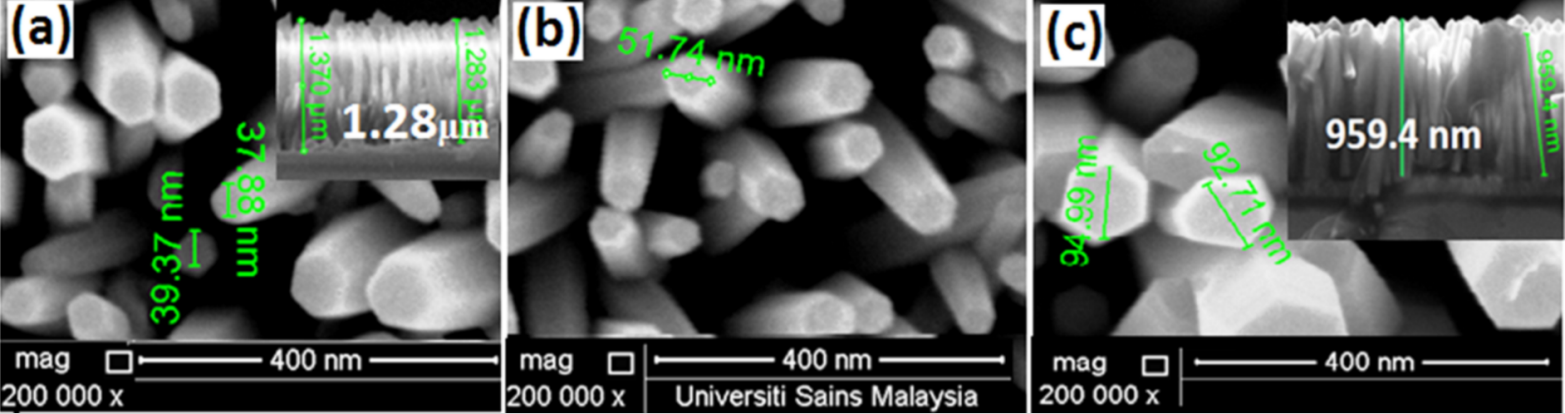
FESEM images with cross-sectional views (inset) of the ZnO nanorods grown on (a) glass, (b) PET and (c) Si substrates at 95 °C for 3 h, respectively.

Figure 2a–c shows magnified FESEM images of the fabricated ZnO nanorods along with the corresponding cross-sectional views. The images reveal the formation of vertically oriented nanorods with various aspect ratios. For example, the nanorods grown on the glass substrates (Figure 2a) have an average length of 1200 nm and a diameter of 38 nm, while those grown on PET substrates (Figure 2b) have an average diameter of 51 nm and a length of 1180 nm. The nanorods grown on Si substrates (Figure 2c) were found to have an average length of 960 nm and a diameter of 92 nm. This implies that the aspect ratio of the single-crystal ZnO nanorods along the (002) direction is sensitive to the substrate type. This can be related to the different mismatch between each substrate and the ZnO seed layer.

**Figure 3:**
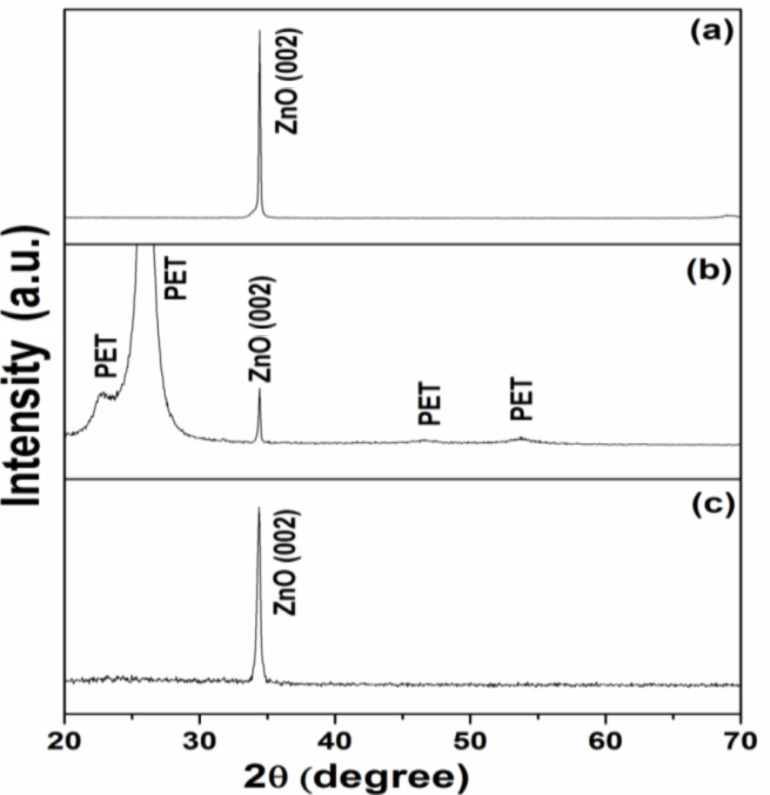
Typical XRD patterns of the ZnO nanorod arrays grown at 95 °C for 3 h on (a) Si, (b) PET and (c) glass substrates, respectively.

Figure 3a–c shows a typical XRD pattern for the ZnO nanorod arrays grown on Si, PET and glass substrates, respectively. For all of the tested ZnO nanorods, a peak is observed at 2θ = 34.4 ± 0.03° corresponding to ZnO (002), revealing the preferentially oriented growth along the *c*-axis. All diffraction peaks are consistent with the wurtzite structure of ZnO [16], which can be indexed to a standard spectrum in the ICSD database (No. 01-079-0207). Additional broad and highly intense diffraction peaks were observed at a 2θ of 25.9° and 22.7° (and with less intensity at a 2θ of 46.5° and 55.1°) for the nanorods grown on a PET substrate, which can be related to the PET substrate itself.

The lattice constant *c* along the preferentially oriented growth direction for the hexagonal crystal structure can be calculated using Equation 1, where *d* is the distance between adjacent planes with Miller–Bravais indices (*hkl*), and *a* and *c* are the lattice constants [16].

[1]
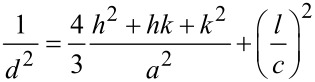


The resulting values of *c* are listed in Table 1. The variation of these values can be attributed to the strain on the ZnO nanorods due to thermal and lattice mismatching between the substrates and ZnO. The Scherer equation (Equation 2) was used to evaluate the average crystallite size (*D*) of ZnO from the dominant (002) growth orientation, where β is the full width at half maximum (FWHM) intensity, *k* is a constant (0.90), and λ is the incident X-ray wavelength [16].

[2]
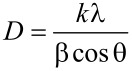


The obtained results reveal that the increase in the crystallite size is associated with the growth conditions and the type of substrate. For example, the crystallite sizes were 42.230, 42.227 and 33.813 nm for the ZnO nanorods grown on Si, PET and glass substrates, respectively. This variation in crystallite size can clearly be observed in the XRD pattern shown in Figure 3 as a decrease in the FWHM of the ZnO peaks. The dependence of the *D* values on the type of substrate can be attributed to both lattice and thermal mismatching between these substrates and ZnO. As reported in previous work [17], the lattice and thermal mismatches between ZnO and a Si substrate are 15 and 60%, respectively. PET and glass substrates are amorphous, and are thus expected to result in even larger lattice and thermal mismatching with ZnO than for the Si substrate [18]. In the current study, the lattice mismatch is more effective and can be considered as the main reason for the higher *D* values and minimized perpendicular strain along the *a*-axis (ε_a_) as compared to that of Si. The perpendicular strain (ε_a_) can be calculated using Equation 3 [19], where *a* is the calculated lattice parameter and *a*_0_ is the corresponding unstrained values of the lattice parameter.

[3]
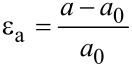


According to the ICSD diffraction data (No. 01-079-0207), *a*_0_ = 5.2125 Å. The values of ε_a_ are positive for the tensile strain and negative for the compressive strain. The values of the obtained *a*, ε_a_ and D are listed in Table 1, indicating compressive strain.

**Table 1:** Lattice parameter (*a*), strain (ε_a_) and average crystallite size (*D*) calculated for ZnO nanorods grown on different substrates.

ZnO /substrate	2θ	*a* (Å )	*d* (Å)	FWHM	*D* (nm)	ε_a_ (%)

ZnO/Si	34.435	3.008	2.605	0.197	42.230	−7.286
ZnO/PET	34.420	3.007	2.606	0.197	42.227	−7.448
ZnO/glass	34.374	3.004	2.609	0.246	33.813	−7.739

Wurtzite ZnO belongs to the *C*_6_*_v_*^4^ (*P*63*mc*) space group, having two formula units in the primitive cell with all atoms occupying *C*_3_*_v_*. Also, wurtzite ZnO has eight sets of optical phonon modes at the Brillion zone center (*q* = 0). These include the Raman active Г = 2 × (A_1_ + B_1_ + E_1_ + E_2_) phonon modes, acoustic modes with A_acoustic_ = A_1_ + E, and optical modes with O_optical_ = A_1_ + (2 × B_1_) + E_1_ + (2 × E_1_). The A and E modes are polar and split into transverse optical (TO) and longitudinal optical (LO) phonons while the B modes are silent modes. According to the selection rules, E_1_, E_2_ and LO are Raman active, while the E and TO are forbidden. Due to the long-range electrostatic forces, the phonons with A_1_ and E symmetry are polar and hence exhibit different frequencies for the TO and LO phonons. Every mode corresponds to a band in the Raman spectrum, with the A_1_ phonon vibration polarized parallel to the *c*-axis and the E phonon polarized perpendicular to the *c*-axis. The intensities of these bands depend on the scattering cross section of these modes [20].

**Figure 4:**
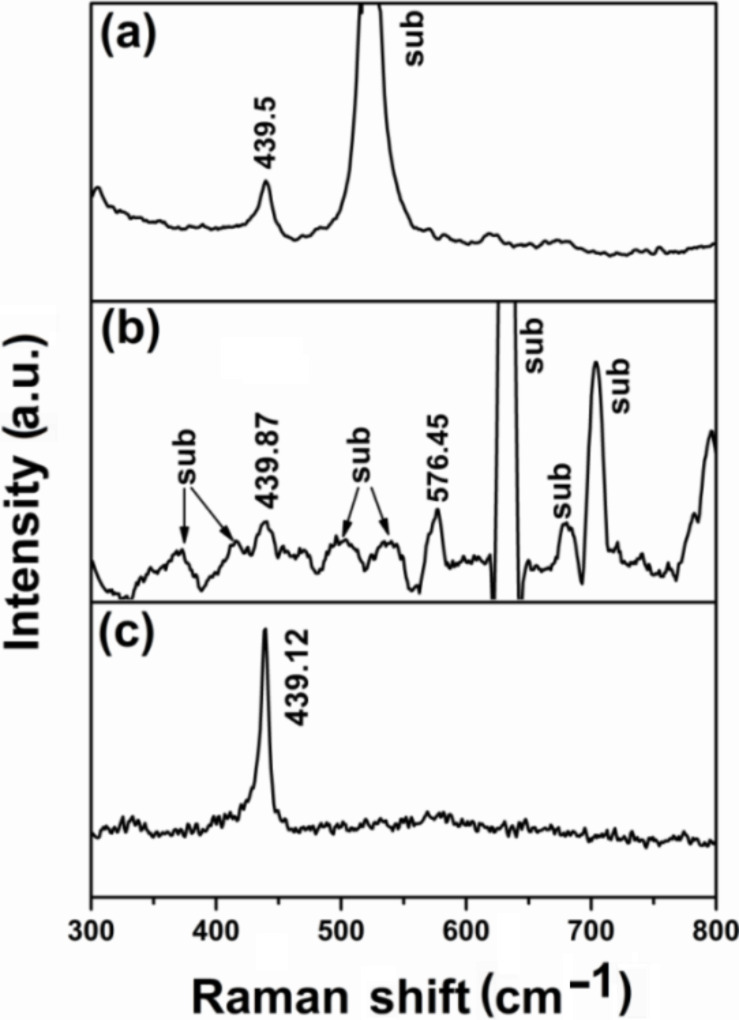
Typical Raman spectra of the ZnO nanorods grown at 95 °C for 3 h on (a) glass, (b) PET and (c) Si substrates.

Figure 4a–c shows the obtained Raman spectra of the single-crystal ZnO nanorod arrays grown on Si, PET and glass substrates, respectively. All samples exhibit the same peaks but with slightly different Raman shifts. The peak at ≈439 cm^−1^ is attributed to the E_2_ (high) mode, which is shifted by 2.6 cm^−1^ compared to the standard E_2_ (high) phonon mode in standard unstrained ZnO samples (437.0 cm^−1^). This shift can be attributed to the compressive stress that exists in the ZnO nanorod samples, in good agreement with previous reports [21–22]. The peak at 576.45 cm^−1^ in sample (b) (PET substrate) is an intrinsic LO mode of hexagonal wurtzite ZnO [23]. This peak is a result of the combination of resonance at the excitation wavelength and impurity-induced scattering [22]. Also, other highly intense peaks were detected in sample (b) in the region 600–890 cm^−1^ characteristic of the PET substrate (sub), which also can be seen in sample (a) for glass substrate (sub), yet it was not detected for the Si substrate.

## Conclusion

Single-crystal ZnO nanorod arrays were successfully synthesized on glass, PET and Si substrates by a cost-effective, optimized CBD method. The results indicate the possibility to grow single-crystal ZnO nanorods in a predominantly (002) growth orientation on different substrates by adjusting the deposition conditions. The aspect ratio of the fabricated single-crystal ZnO nanorods was found to be sensitive to the substrate type. The nanorods grown on glass substrates showed an average length of 1200 nm and a diameter of 38 nm, while those grown on PET substrates had an average diameter of 51 nm and a length of 1180 nm. The nanorods grown on Si substrates were found to have an average length of 960 nm and diameter of 92 nm. The calculated crystallite size revealed that the increase in the crystallite size is strongly associated with the growth conditions with a minor dependence on the type of the substrate. The Raman spectra measurements showed the same peaks for all of the samples with only a small variation at 439 cm^−1^, corresponding to the E_2_ (high) mode. This shift towards higher wavenumbers (blue shift) can be attributed to the compressive stress effects.
